# Isoliquiritigenin Inhibits Ovarian Cancer Metastasis by Reversing Epithelial-to-Mesenchymal Transition

**DOI:** 10.3390/molecules24203725

**Published:** 2019-10-16

**Authors:** Chen Chen, Shuang Huang, Chang-Liang Chen, Shi-Bing Su, Dong-Dong Fang

**Affiliations:** 1 Institute of Interdisciplinary Integrative Medicine Research, Shanghai University of Traditional Chinese Medicine, Shanghai 201203, China; joyce5258@126.com; 2 Department of Anatomy and Cell Biology, University of Florida College of Medicine, 2033 Mowry Road, P.O. Box 103633, Gainesville, FL 32610, USA; shuanghuang@ufl.edu; 3 Department of Obstetrics and Gynecology, Medical College of Wisconsin, 8701 W Watertown Plank Rd, Wauwatosa, WI 53226, USA; cclmlt1987@gmail.com

**Keywords:** isoliquiritigenin, epithelial-to-mesenchymal transition, metastasis, ovarian cancer

## Abstract

The epithelial-to-mesenchymal transition (EMT) plays a prominent role in cancer metastasis. Isoliquiritigenin (ISL), one of the flavonoids in licorice, has been shown to exhibit anticancer activities in many cancer types through various mechanisms. However, it is unknown whether ISL impacts the EMT process. Here, we show that ISL is able to suppress mesenchymal features of ovarian cancer SKOV3 and OVCAR5 cells, evidenced by an apparent morphological change from a mesenchymal to an epithelial phenotype and reduced levels of mesenchymal markers accompanied by the gain of E-cadherin expression. The suppression of EMT is also supported by the observed decrease in cell migration and in vitro invasion upon ISL treatment. Moreover, we show that ISL effectively blocks the intraperitoneal xenograft development of the SKOV3 cell line and prolonged the survival of tumor-bearing mice. These data suggest that ISL inhibits intraperitoneal ovary tumor development through the suppression of EMT, indicating that ISL may be an effective therapeutic agent against ovarian cancer.

## 1. Introduction

Ovarian cancer is the most lethal gynecologic cancer [1,2]. As early symptoms are nonspecific and difficult to detect, 70% of women with ovarian cancer are diagnosed with metastasis and at an advanced stage [1,2]. The main treatment for ovarian cancer is surgery to remove the diseased tissue followed by chemotherapy [3]. Although most patients respond initially, almost all of them relapse and ultimately die due to metastasis [1]. Therefore, finding ways to block metastasis may be the most effective therapeutic strategy to treat ovarian cancer.

Extensive evidence supports the role of epithelial-to-mesenchymal transition (EMT) in cancer metastasis [4,5,6]. EMT is a process by which epithelial cells lose their polarity and gain invasive properties to become mesenchymal cells [7,8,9]. During EMT, the expression of epithelial markers such as E-cadherin is downregulated, whereas the expression of mesenchymal markers, including N-cadherin and vimentin, is upregulated [7,8,9]. The EMT process is controlled through the activation of EMT-associated transcriptional factors, including ZEB1, ZEB2, and Twist1 [7,8,9].

The roots of licorice (*Glycyrrhiza*) have been customarily used in traditional Chinese medicine and as natural sweeteners. Isoliquiritigenin (2′-4′-4-trihydroxychalcone, ISL), which has a chalcone structure [10] and is isolated from the root of licorice, is considered to be the main biologically active component for the useful pharmacological properties of licorice roots, such as their anti-inflammatory [11,12], antioxidative [13], antiviral [14], anti-platelet-aggregation [15], hepatoprotective [16], immunoregulatory [17], and cardioprotective effects [18]. Apart from these basic effects, ISL exhibits many anticancer activities [19,20,21]. For example, ISL has been reported to induce apoptosis and autophagy in breast cancer [22], lung cancer [23,24], and hepatocellular carcinoma (HCC) [25]. ISL has also been shown to inhibit breast and lung cancer cell migration [26,27,28]. We previously showed that ISL strongly deterred lung cancer cell migration and tumorigenicities by interfering with the Src signaling pathway through its metabolite tetrahydroxychalcone (THC) [29]. In several recent reports, ISL was found to block cell growth through the induction of apoptosis and autophagy in ovarian cancer cells [30,31]. Given the prominent role of Src in EMT, it is of great interest to investigate whether ISL can suppress ovarian cancer EMT and metastasis.

In the present study, we observed that ISL, at a noncytotoxic concentration, was able to suppress EMT traits in ovarian cancer cells. Importantly, we demonstrated that ISL impeded intraperitoneal xenograft development and prolonged the life span of tumor-bearing mice.

## 2. Results

### 2.1. ISL at a Noncytotoxic Concentration Suppresses Ovarian Cancer Cell EMT Traits

**Figure 1:**
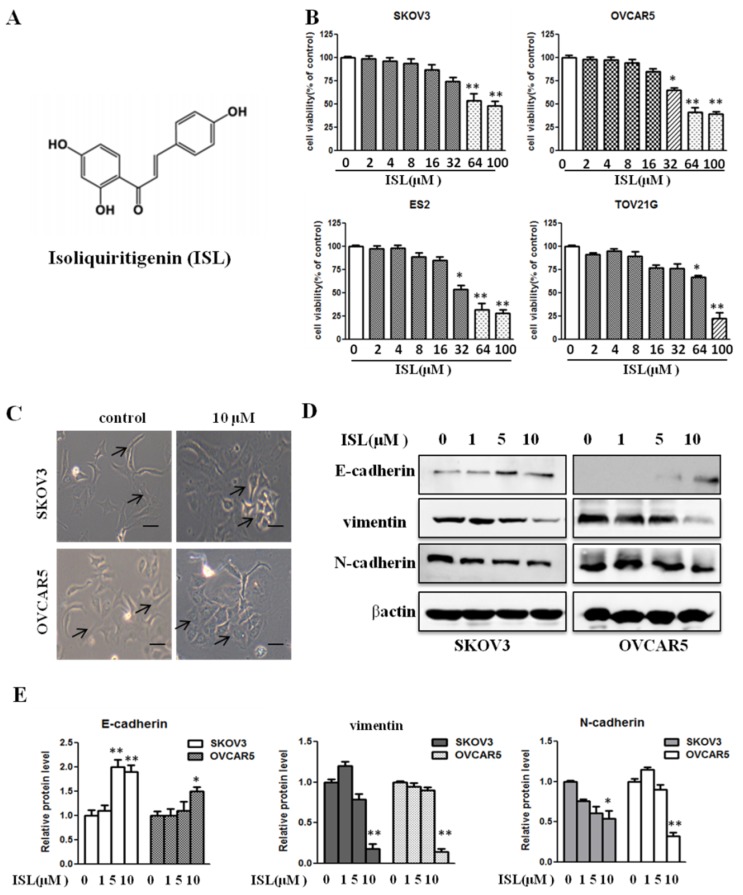
Isoliquiritigenin (ISL) at a noncytotoxic concentration suppresses ovarian cancer cell epithelial-to-mesenchymal transition (EMT) traits. (**A**) Chemical structure of ISL. (**B**) The cell viability of SKOV3, OVCAR5, ES2, and TOV21G cells treated with ISL (2, 4, 8, 16, 32, 64, and 100 μM) for 72 h was determined by 3-(4,5-dimethylthiazol-2-yl)-2,5-diphenyltetrazolium bromide (MTT) assay. (**C**) Observed cell morphology of cells treated with ISL (10 μM) for 72 h; 100× magnification. Scale bars, 25 μm. (**D**) Cells were treated with ISL (1, 5, and 10 μM) for 72 h and analyzed by Western blotting. (**E**) Bar graph shows results of quantitative analysis of Western blotting. Protein expression is presented as fold changes and normalized to β-actin. Data are presented as mean ± SD, *n* = 3. Student *t*-test was used for statistical tests. * *p* < 0.05, ** *p* < 0.01 compared with the control group.

ISL has been reported to induce apoptosis in various cancer cell types [22,23,24,25]. We investigated whether ISL could similarly reduce cell viability in a panel of ovarian cancer cell lines, including mesenchymal-like SKOV3, OVCAR5, and ES2 as well as epithelial-like TOV21G cells, by culturing them in the absence or presence of various concentrations of ISL (2, 4, 8, 16, 32, 64, and 100 μM) for 72 h. With the aid of a 3-(4,5-dimethylthiazol-2-yl)-2,5-diphenyltetrazolium bromide (MTT) assay, we observed that a low concentration of ISL had no significant effect on cell viability, although concentrations at 64 and above 100 μM were growth inhibitory in all four lines (Figure 1B). To determine whether ISL impacts EMT traits of ovarian cancer cells, we treated SKOV3 and OVCAR5 cells with ISL at noncytotoxic concentrations (1, 5, and 10 μM) for 72 h. Microscopic analyses revealed that 10 μM of ISL led to a morphological change from an elongated mesenchymal to a cobblestone-shaped epithelial phenotype in both cell lines (Figure 1C).

To substantiate the potential EMT-suppressive role of ISL, we further examined the abundance of EMT markers in SKOV3 and OVCAR5 cells treated with ISL (1, 5, and 10 μM). A Western blot assay showed that 10 μM of ISL increased the level of epithelial marker E-cadherin and reduced the amount of the mesenchymal markers vimentin and N-cadherin (Figure 1D,E). These results suggest that ISL possesses the capacity to suppress EMT in ovarian cancer cells.

### 2.2. ISL Inhibited SKOV3 and OVCAR5 Migration and Invasion

**Figure 2:**
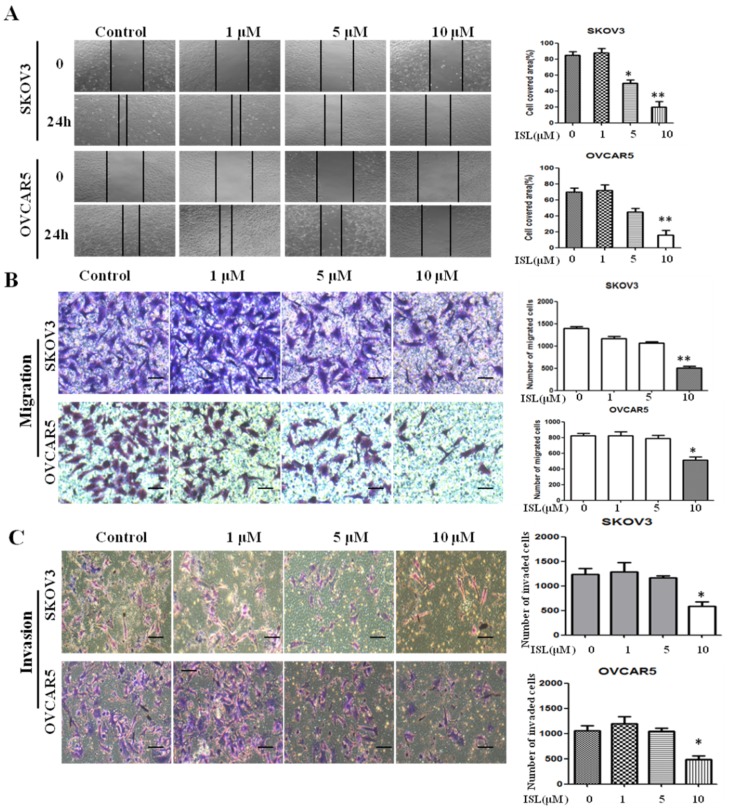
ISL inhibits the migration and invasion of SKOV3 and OVCAR5 cells. (**A**) Cells were grown to confluence, followed by treatment with vehicle or ISL (1, 5, and 10 μM) for 24 h. A scratch was made with a fine pipette tip and cells were kept in medium containing 2% FBS with or without ISL. Images were taken at 0 and 24 h under a phase-contrast microscope. (**B**) Photomicrographs of migration to the lower side of chamber. Cells were pretreated with vehicle or ISL (1, 5, and 10 μM) for 48 h, followed by analysis of cell migration using a transwell assay. Bar graph shows results of quantitative analysis of migration. The number of stained cells in five randomly selected fields was counted. (**C**) Photomicrographs of invasion to the lower side of chamber. Cells were pretreated with vehicle or ISL (1, 5, and 10 μM) for 48 h, followed by analysis with a cell Matrigel invasion assay. Bar graph shows the results of quantitative analysis of invasion. The number of stained cells in five randomly selected fields was counted. Data are presented as mean ± SD. *n* = 3. 100× magnification. Scale bars, 25 μm. Student *t*-test was used for statistical tests. * *p* < 0.05, ** *p* < 0.01 compared with the control group.

As robust EMT occurrence always accompanies increased cell migration and invasion, we hypothesized that ISL is an effective agent to deter these features in mesenchymal-like ovarian cancer cells. To test this hypothesis, we initially performed a wound-healing assay to assess the effect of 10 µM of ISL on cell migration. While the gaps were nearly filled at 24 h in vehicle-treated SKOV3 or OVCAR5 cells, they were barely filled in ISL-treated cells (Figure 2A). Further, a transwell assay similarly showed that cells previously exposed to 10 µM of ISL for 48 h migrated much slower than cells exposed only to vehicle (Figure 2B). Subsequently, we utilized Matrigel invasion chambers to evaluate the effect of ISL on the in vitro invasion of SKOV3 and OVCAR5 cells. Cells pretreated with 10 µM of ISL for 48 h displayed greatly reduced invasion compared with those treated with vehicle (Figure 2C). These results are consistent with the notion that ISL can effectively suppress EMT in ovarian cancer cells.

### 2.3. ISL Downregulated the Expression of EMT-Associated Transcription Factor ZEB1

**Figure 3:**
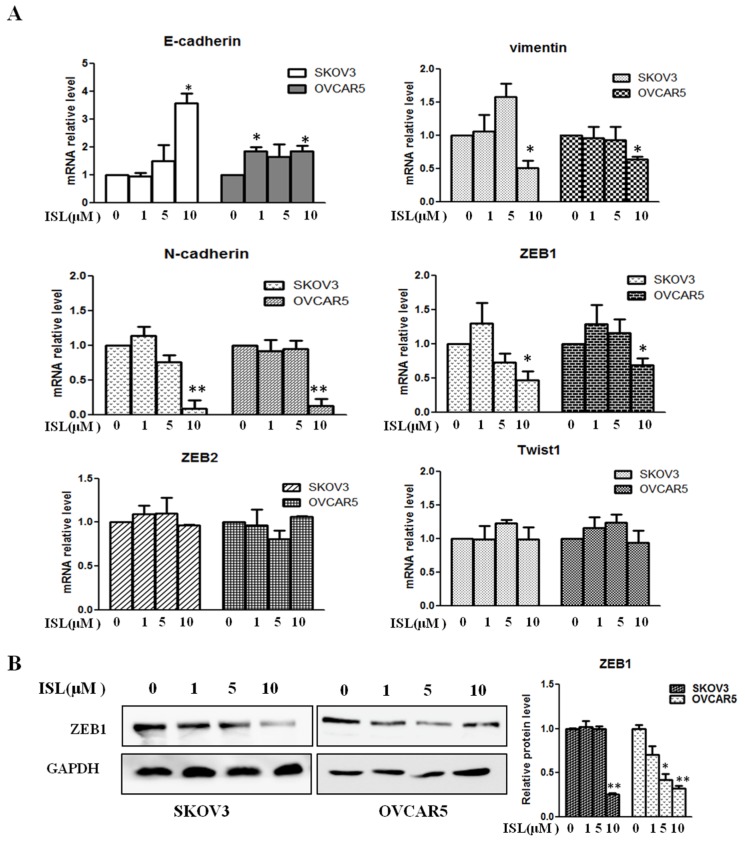
Effects of ISL on the expression of E-cadherin, Vim, N-cadherin, Twist1, ZEB1, and ZEB2 in SKOV3 and OVCAR5 cells. (**A**) Cells were treated with vehicle or ISL (1, 5, and 10 μM) for 72 h, followed by qRT-PCR analysis. Gene expression is presented as fold changes and normalized to β-actin. (**B**) Cells were treated with ISL (1, 5, and 10 μM) for 72 h and analyzed by Western blotting. The bar graph shows results of a quantitative data of Western blotting. Protein expression is presented as fold changes and normalized to GAPDH. Data are presented as mean ± SD. *n* = 3. Student *t*-test was used for statistical tests. * *p* < 0.05, ** *p* < 0.01 compared with the control group.

To elucidate the molecular mechanism by which ISL suppresses EMT in ovarian cancer cells, we performed an expression array to assess the changes in the mRNA levels of 84 EMT-associated genes between untreated and ISL-treated SKOV3 cells. Among those factors with a significant reduction in their mRNA levels, we noticed that EMT-associated transcription factors ZEB1 and ZEB2 were much lower in ISL-treated SKOV3 cells than untreated ones (Table 1), further indicating the deterrence of EMT by ISL in ovarian cancer cells. To validate the findings, we carried out a qRT-PCR assay to measure the levels of ZEB1 and ZEB2 along with E-cadherin and vimentin mRNA. The expression of N-cadherin and Twist1 were also confirmed by qRT-PCR assay. While 10 μM of ISL led to an increase in E-cadherin and a decrease in vimentin and N-cadherin mRNA, we observed a dramatic decrease in the level of ZEB1 mRNA, although the level of ZEB2 and Twist1 mRNA was unchanged by ISL (Figure 3A). Further, Western blotting also showed that ISL reduced the amount of ZEB1 protein in both SKOV3 and OVCAR5 cells (Figure 3B). These results suggest that downregulation of ZEB1 is at least one of the mechanisms contributing to ISL-led inhibition in ovarian cancer EMT.

An expression array to assess the change in the levels of 84 EMT-associated genes using total RNA isolated from untreated and ISL-treated SKOV3 cells. Compared with the control, 55 genes decreased, 12 increased, and 14 unchanged in ISL-treated SKOV3 cells.

### 2.4. ISL Blocked Intraperitoneal Xenograft Development and Prolonged the Survival of Ovary-Tumor-Bearing Mice

**Figure 4:**
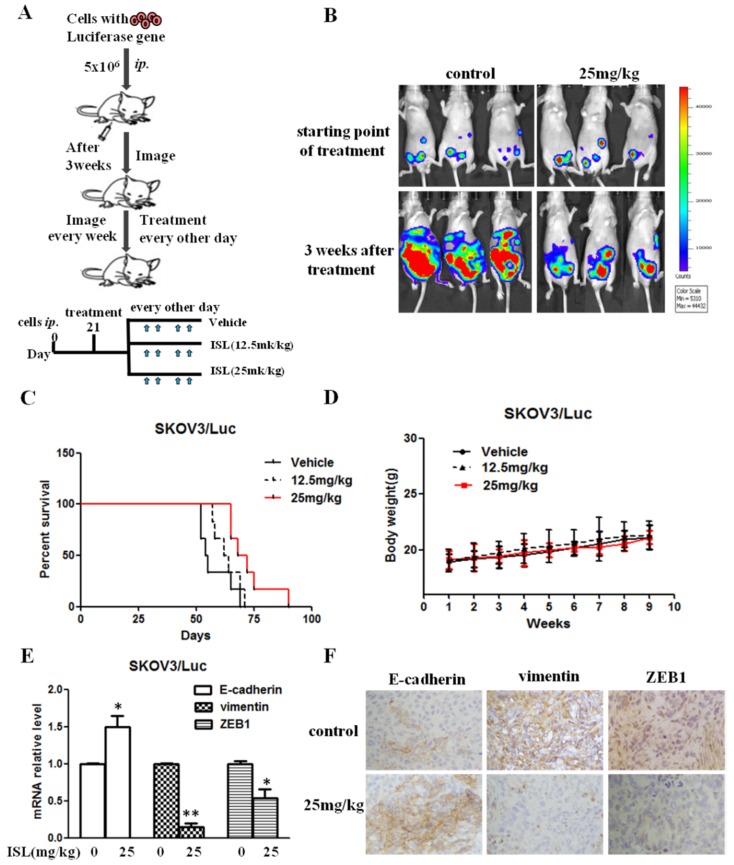
ISL blocked intraperitoneal xenograft development and prolonged the survival of ovary-tumor-bearing mice. (**A**) Flowchart of therapy scheme. (**B**) Luciferase-containing SKOV3 cells (5 × 10^6^ cells/mouse) were injected intraperitoneally into nude mice for 3 weeks, followed by administration of 12.5 or 25 mg/kg of ISL. Tumors were imaged in the Xenogen system. (**C**) Kaplan–Meier analysis of animal endpoint survival following treatment with ISL. (**D**) Body weight was measured every week. (**E**) The mRNA expression of E-cad, Vim, and ZEB1 was detected in tumor tissues. Gene expression is presented as fold changes and normalized to β-actin. (**F**) Representative pictures of immunohistochemical staining of E-cadherin, Vim, and ZEB1 in tumor tissues. Scale bars, 20 μm. Data are presented as mean ± SD, *n* = 6. Student *t*-test was used for statistical tests. * *p* < 0.05, ** *p* < 0.01 compared with the vehicle group.

We previously reported that EMT status is associated with the ability of ovarian cancer cells to undergo intraperitoneal xenograft development [32,33]. The observation that ISL effectively blocked ovarian cancer EMT prompted us to investigate the effect of ISL on intraperitoneal xenograft development of ovarian cancer cells (the therapeutic scheme is depicted in Figure 4A). Female athymic nude mice were intraperitoneally injected with luciferase-expressing SKOV3 cells for 3 weeks, followed by intraperitoneal administration of ISL. Bioluminescence imaging showed that intraperitoneal xenografts were detected 3 weeks after ovarian cancer cell injection (Figure 4B). Compared with the vehicle group, administering ISL (25 mg/kg) deterred tumor development (Figure 4B) and prolonged the survival of tumor-bearing mice (Figure 4C). The body weights of ISL-treated groups were approximately the same as those of the vehicle group (Figure 4D). To link ISL-led suppression in xenograft development to blocked EMT traits, we analyzed the expression of E-Cad, Vim, and ZEB1 in the collected tumors. QRT-PCR showed the elevated E-cadherin but reduced Vim and ZEB1 mRNA in tumors excised from ISL-treated mice compared with those from the control group (vehicle treated) (Figure 4E). Immunohistochemistry (IHC) analysis further showed stronger E-cadherin staining but weaker Vim and ZEB1 staining in ISL-treated groups compared to the control group (Figure 4F). These results suggest that ISL suppresses intraperitoneal xenograft development of ovarian cancer cells by blocking EMT.

## 3. Discussion

Ovarian cancer is the most lethal gynecologic malignancy and is a highly metastatic disease [1]. Unfortunately, the early stage of ovarian cancer is hard to detect, and reliable screening tools have yet to be developed [2,3]. Since most ovarian cancers are detected only after the cancers have spread to other organs [2,3], a promising strategy to combat the disease is to control ovarian cancer metastasis. Although there is some controversy over the general role of EMT in cancer metastasis, extensive evidence from ovarian cancer studies support the notion that EMT is critical for ovarian cancer metastasis [4,5,6]. We posit that effective anti-ovarian cancer therapeutic approaches can be established by deterring EMT in ovarian cancer. We have previously shown that ISL, a flavonoid compound, is a potent Src inhibitor [29], and the Src signaling pathway is critical for the maintenance of EMT traits in ovarian cancer cells [33]. This raises the possibility that ISL may possess the capacity to block ovarian cancer EMT and subsequent peritoneal metastasis.

In this study, we found that ISL was only able to decrease ovarian cancer cell growth at a concentration that was >32 μM, which is consistent with previous reports in which ISL was shown to induce cell death at a similar concentration [30,31]. Interestingly, ISL was able to block ovarian cancer cell migration and invasion at 10 µM, indicating that distinct cellular mechanisms are responsible for ISL-led inhibition of cell growth and migration/invasion. In fact, we found that ISL at a noncytotoxic concentration was able to antagonize EMT and block intraperitoneal xenograft development of ovarian cancer cells.

EMT and its reverse process, mesenchymal-to-epithelial transition (MET), are critical for cancer metastasis [4,5,6]. The typical features of MET are the gain of epithelial cell junction proteins, such as E-cadherin, and the loss of mesenchymal markers, such as vimentin and N-cadherin, accompanied by the switch of cell morphology from a mesenchymal to an epithelial phenotype. We noticed that ISL at a noncytotoxic concentration led to a morphological change from a mesenchymal to an epithelial shape in both SKOV3 and OVCAR5 cells. Similarly, we observed increased levels of the epithelial marker E-cadherin and reduced amounts of mesenchymal markers, including vimentin and N-cadherin, upon ISL treatment, indicating that ISL at a noncytotoxic concentration led to a robust MET in ovarian cancer cells. Although mRNA expression of vimentin seems to be increased by 5 μM of ISL treatment, there was no statistical significance. Additionally, E-cadherin expression did not significantly change by same dose of ISL. In line with the well-recognized role of EMT in cell migration and invasion, we showed that ISL decreased ovarian cancer cell migration and invasion. Importantly, administration of ISL (25 mg/kg) suppressed intraperitoneal xenograft development and extended the life span of tumor-bearing mice.

EMT is regulated by a set of transcriptional factors, including Snail, slug, Twist, ZEB1, and ZEB2. To determine whether ISL deterred EMT by impacting the expression of these transcription factors, we showed that ISL decreased expression of ZEB1. IHC analysis of xenografts also showed reduced ZEB1 staining along with diminished vimentin but increased E-cadherin staining in tumors collected from ISL-treated mice compared with control. Collectively, these findings suggest that ISL inhibited ovarian cancer EMT and intraperitoneal xenograft development through the inhibition of ZEB1. A recent study reported that Notch and TGF-β form a reciprocal positive regulatory loop to cooperatively regulate EMT and promote epithelial ovarian cancer (EOC) cell motility and migration [34]. Another report showed reduced EMT in ovarian cancer cells upon the inhibition of TGF-β1 signaling [35]. In fact, our EMT expression array revealed that ISL reduced the level of TGF-β in ovarian cancer cells. It will be of great interest to investigate whether ISL blocks ovarian cancer EMT by interfering with the TGF-β pathway.

## 4. Materials and Methods

### 4.1. Cells and Other Reagents

All ovarian cancer cells were gifted from Dr. Shuang Huang, Professor, University of Florida College of Medicine, Gainesville, USA. All cells were cultured in DMEM with 10% fetal bovine serum (GIBCO) and incubated in a humidified atmosphere of 95% air and 5% CO_2_ at 37 °C. MTT was purchased from Sigma Aldrich (St. Louis, MO, USA). Isoliquiritigenin was purchased from Shanghai Tongtian Biotechnology Co., Ltd. Information for primer sequences for PCR are included in the Supplementary Information.

### 4.2. Cell Viability Assay

To explore the effect of ISL on ovarian cancer cell growth, we performed the MTT assay as described previously [36]. Briefly, 5 × 10^3^ cells were seeded into a 24-well plate (Corning) and allowed to adhere overnight. Subsequently, cells were replaced with media containing different concentrations of ISL. Control cells were treated similarly, except that there was media in the absence of ISL. After incubation for 72 h, 100 μL/well of MTT solution (0.5 mg/mL) was added to each well and incubated for 2–4 h at 37 °C. The supernatants were discarded. The resulting formazan was dissolved in 500 μL dimethyl sulfoxide (DMSO) and measured by absorbance at 570 nm using a microplate reader (Bio-Rad). Cell viability under each condition is expressed as a percentage of the control, which was set as 100%. 

### 4.3. Cell Morphology Observations

SKOV3 and OVCAR5 cells were seeded in 3.5 cm dishes, incubated overnight, and treated with ISL for 72 h. Cell morphology was captured by a bright-field microscope (Olympus, Hamburg, Germany).

### 4.4. Wound-Healing Assay

Cells were seeded in a 24-well plate and grown to confluence overnight. A scratch was generated with a thin pipette tip. Cells were washed with serum-free medium to remove dislodged cells and then incubated in medium in the absence or presence of ISL for 24 h (gaps were usually filled by approximately 95% at this time point in untreated cells). Cells were photographed using a bright-field microscope (Olympus, Hamburg, Germany) before treatment and at 24 h of incubation. 

### 4.5. Cell Migration and Invasion Assays

Cell migration and invasion assays were performed by using 24-well transwell chambers (8.0 μm pore size) and Matrigel invasion chambers, respectively, as described previously [36,37,38]. Briefly, SKOV3 and OVCAR5 cells were seeded in 3.5 cm dishes and treated with or without ISL for 48 h. Then, pretreated SKOV3 (3 × 10^4^ cells) or OVCAR5 (1 × 10^5^ cells) cells were resuspended in 100 μL of fresh medium containing 1% FBS and seeded into the upper chamber. The transwell chambers were then placed into 24-well plates, into which we added the 500 μL of 10% FBS. After 24 h, the media from the wells were withdrawn. Nonmigratory cells on the upper membrane were gently wiped with cotton swabs, and the cells attached on undersurface were fixed with 4% paraformaldehyde and stained with crystal violet. The number of stained cells in five randomly selected fields was counted. For the invasion assay, cells were pretreated the same way as the cell migration assay. After pretreatment, cells were added to chambers (10^5^ cells in 100 μL/well) and allowed to invade for 24 h.

### 4.6. Real-Time PCR-Based Microarray Assay and qRT-PCR

The effect of ISL on the expression genes associated with EMT was detected by a human EMT RT2 profiler PCR array (Qiagen, Frederick, MD, USA). Total RNA isolated from control SKOV3 cells and ISL-treated SKOV3 cells was used for screening according to the manufacturer’s instructions. For qRT-PCR, total RNA was isolated from cultured cell lines or frozen tumor samples using TRIzol reagent (Invitrogen, Carlsbad, CA, USA) and then reverse transcribed to cDNA using PrimeScript RT reagent kit (Takara, Dalian, Shanghai). The generated cDNA was subjected to quantitative PCR with specific primer sets and amplified using SYBR green Supermix with Rox dye (Applied Biosystems, Waltham, MA, USA). Quantitative PCR was carried out on a StepOnePlus real-time PCR system (Applied Biosystems, USA). The expression levels were standardized by comparing the Ct values of the target to that of β-actin mRNA.

### 4.7. Western Blot Analysis

Cells lysates were prepared and Western blot analyses were performed as previously described [39,40]. Briefly, SKOV3 and OVCAR5 cells were treated with ISL for 72 h and the cell lysates were collected. Total protein was quantitated with the BCA protein assay reagent kit (Biyuntian, Shanghai, China). Equal amounts of protein were separated on SDS-PAGE 10% gels and transferred onto nitrocellulose membranes, incubated (overnight, 4 °C) with primary antibodies, followed by horseradish-peroxidase-conjugated secondary antibodies. Detection was performed using an ECL system (4200SF, Tanon, Shanghai). Primary antibodies for E-cadherin (1:1000), Vim (1:1000), N-cadherin (1:1000), ZEB1 (1:1000), and β-actin (1:1000) were obtained from Cell Signaling Technology (Beverly, MA, USA). Horseradish-peroxidase-linked anti-rabbit IgG (Cell Signaling Technology) was used as a secondary antibody. Immunoreactive proteins on the membrane were visualized by SuperSignal West Femto Enhancer Solution (Thermo, Waltham, MA, USA).

### 4.8. Intraperitoneal Xenograft Development Assay

Six-week-old athymic female nude mice were obtained from Shanghai Laboratory Animal Center (Shanghai, China). Intraperitoneal xenograft development was assessed as previously described [32,33,41]. Briefly, to determine the effect of ISL on metastatic colonization, the mice were first injected with luciferase-expressing SKOV3 cells (5 × 10^6^ cells per mouse) for 3 weeks. ISL dissolved in 0.5% hydroxypropyl methylcellulose/0.1% Tween-80 were administered intraperitoneally to the mice with the aid of gavages every other day. Doses for ISL were 12.5 and 25 mg/kg, respectively. The control mice were given vehicle every other day. Tumor progression was monitored by examining fluorescence in a Xenogen IVIS-200 In Vivo Imaging System on a weekly basis as previously described [29,33]. Visible implants in peritoneal cavities were harvested and fixed for IHC and qRT-PCR.

### 4.9. Immunohistochemistry

Tumor tissues were collected from tumor-bearing mice immediately postmortem. Paraffin-embedded tissues were sectioned and subjected to IHC to detect E-cadherin, Vim, and ZEB1 using the respective antibodies as previously described [33].

### 4.10. Statistical Analysis

Quantitative data are expressed as mean ± SD. Comparisons were analyzed by Student *t*-test. All data are the results of at least three independent experiments.

### 4.11. Ethics Approval and Consent to Participate

All animal procedures were conducted in accordance with the guidelines of the National Institutes of Health and were approved by the Ethical Committee of the Shanghai University of Traditional Chinese Medicine (Approval ID: PZSHUTCM190628021).

## 5. Conclusions

In conclusion, this study provides strong evidence that ISL at a noncytotoxic concentration inhibited EMT, migration, and invasion in SKOV3 and OVCAR5 cells. ISL also reduced the metastasis of ovarian cancer and extended the life span of animals bearing SKOV3/Luc cells by blocking the EMT process and regulating the expression of ZEB1. Our data indicate that ISL may be a promising repressor against the metastasis of ovarian cancer by affecting EMT.

## Figures and Tables

**Table 1:** Expression of EMT-Associated Genes in untreated (control) *vs* ISL-treated SKOV3 cells (ISL).

Gene	Control	ISL	Gene	Control	ISL	Gene	Control	ISL
AHNAK	1	0.734623	IL1RN	1	0.418554	SMAD2	1	0.572358
AKT1	1	0.924291	ILK	1	0.785611	SNAI1	1	1.566291
BMP1	1	0.404673	ITGA5	1	0.535606	SNAI2	1	1.131232
BMP2	1	0.743396	ITGAV	1	0.513642	SNAI3	1	2.443003
BMP7	1	0.31247	ITGB1	1	0.594326	SOX10	1	0.510887
CALD1	1	0.419532	JAG1	1	0.435286	SPARC	1	1.120049
CAMK2N1	1	0.767472	KRT14	1	1.161878	SPP1	1	4.084098
CAV2	1	0.53588	KRT19	1	0.717957	STAT3	1	0.572006
CDH1	1	1.068836	KRT7	1	0.497813	STEAP1	1	0.182371
CDH2	1	0.296297	MAP1B	1	0.413056	TCF3	1	1.037395
COL1A2	1	0.373447	MMP2	1	1.518571	TCF4	1	0.875013
COL3A1	1	0.374667	MMP3	1	0.474877	TFPI2	1	0.403746
COL5A2	1	0.668282	MMP9	1	0.924883	TGFB1	1	0.583945
CTNNB1	1	0.573468	MSN	1	1.18802	TGFB2	1	0.394966
DSC2	1	0.948605	MST1R	1	0.764398	TGFB3	1	1.295126
DSP	1	0.463705	NODAL	1	1.54975	TIMP1	1	1.09639
EGFR	1	0.396217	NOTCH1	1	0.595044	TMEFF1	1	0.619747
ERBB3	1	0.34008	NUDT13	1	0.524084	MEM132A	1	0.890626
ESR1	1	0.696109	OCLN	1	0.350573	TSPAN13	1	1.582411
F11R	1	0.662682	PDGFRB	1	/	TWIST1	1	0.811606
FN1	1	0.797309	PLEK2	1	1.065871	VCAN	1	0.987891
FOXC2	1	0.659119	PPPDE2	1	0.419386	VIM	1	1.056343
FZD7	1	0.748245	PTK2	1	0.654937	VPS13A	1	0.762398
GNG11	1	0.770319	PTP4A1	1	0.389229	WNT11	1	2.137225
GSC	1	1.215896	RAC1	1	0.782444	WNT5A	1	1.517202
GSK3B	1	0.321123	RGS2	1	1.443939	WNT5B	1	2.762547
IGFBP4	1	0.614624	SERPINE1	1	0.598634	ZEB1	1	0.602478
			SIP1	1	0.928877	ZEB2	1	0.506116

## Supplementary Materials

Supplementary materials are available online.

## Abbreviations

EMT	Epithelial-to-mesenchymal transition
ISL	Isoliquiritigenin
Vim	vimentin
ZEB1	Zinc finger E-box binding homeobox 1
qRT-PCR	Real-time quantitative reverse transcription PCR
MTT	3-(4,5-Dimethylthiazol-2-yl)-2,5-diphenyltetrazolium bromide
MET	Mesenchymal-to-epithelial transition
IHC	Immunohistochemistry
TGF-β	Transforming growth factor β
DMSO	Dimethyl sulfoxide
