# Supplementary material for: Isoliquiritigenin Inhibits Ovarian Cancer Metastasis by Reversing Epithelial-to-Mesenchymal Transition
Source: Molecules. 2019 Oct 16;24(20):3725. doi: 10.3390/molecules24203725 (PMC6833095; doi:10.3390/molecules24203725)
Supplement: Supplementary file 1 [file molecules-24-03725-s001.pdf]

SUPPLEMENTARY INFORMATION

Primer sequences for PCR

Vimentin forward 5'- AATGACCGCTTCGCCAACT-3'

Reverse 5'- ATCTTATTCTGCTGCTCCAGGAA-3'

E-cadherin forward 5'- ACAGCCCCGCCTTATGATT-3'

Reverse 5'- TCGGAACCGCTTCCTTCA-3'

Zeb1 forward 5'-CACCATCCCCATCACCTCTAA-3'

reverse 5'-GCACCCTCAGCTGTGTACAAGT-3'

Zeb2 forward 5'-GCCGAGTCCATGCGAACT-3'

Reverse 5'-CCATGATCGGCTGCTTCAT-3'

N-cadherin forward 5'-CCTCCAGAGTTTACTGCCATGAC-3'

Reverse 5'-GTAGGATCTCCGCCACTGATTC-3'

twist1 forward 5'-GCCAGGTACATCGACTTCCTCT-3'

Reverse 5'-TCCATCCTCCAGACCGAGAAGG-3'

β-actin forward 5'- CCAGCT CACCATGGAT GATG-3';

Reverse 5'- GACAAC GGCTCCGGCA T-3'.
